# Recomendações para o fortalecimento da atenção primária à saúde no Brasil

**DOI:** 10.26633/RPSP.2020.4

**Published:** 2020-01-06

**Authors:** Renato Tasca, Adriano Massuda, Wellington Mendes Carvalho, Claudia Buchweitz, Erno Harzheim

**Affiliations:** 1 Organização Pan-Americana da Saúde (OPAS) Organização Pan-Americana da Saúde (OPAS) BrasíliaDF Brasil Organização Pan-Americana da Saúde (OPAS), Brasília (DF), Brasil.; 2 Harvard T.H. Chan School of Public Health Departamento de Saúde Global e Populações BostonMA Estados Unidos da América Harvard T.H. Chan School of Public Health, Departamento de Saúde Global e Populações, Boston (MA), Estados Unidos da América; 3 Consultora independente Consultora independente Porto AlegreRS Brasil Consultora independente, Porto Alegre (RS), Brasil.; 4 Universidade Federal do Rio Grande do Sul (UFRGS) Programa de Pós-Graduação em Epidemiologia Porto AlegreRS Brasil Universidade Federal do Rio Grande do Sul (UFRGS), Programa de Pós-Graduação em Epidemiologia, Porto Alegre (RS), Brasil.

**Keywords:** Atenção primária à saúde, estratégia saúde da família, sistema único de saúde, Brasil, Primary health care, family health strategy, unified health system, Brazil, Atención primaria de salud, estrategia de salud familiar, sistema único de salud, Brasil

## Abstract

**Objetivo.:**

Elaborar recomendações estratégicas para fortalecer a atenção primária à saúde (APS) no Sistema Único de Saúde (SUS) no Brasil a partir da consulta a especialistas.

**Método.:**

Este estudo qualitativo, desenvolvido de março a agosto de 2018, foi composto pela aplicação de questionário aberto e construção de consenso entre 20 participantes representativos das cinco macrorregiões brasileiras, selecionados a partir do critério de reconhecida experiência profissional na APS. Os participantes responderam 20 perguntas abertas em questionário *on-line* elaborado pelos pesquisadores. Os achados foram sistematizados na forma de recomendações, submetidas a avaliação de prioridade pelo grupo de especialistas utilizando a metodologia Delphi em rodada única. As recomendações finais foram discutidas em oficina presencial.

**Resultados.:**

Dos 20 especialistas, 18 responderam ao questionário aberto, gerando 84 temas, sistematizados em 44 propostas. Após avaliação, foram elaboradas 20 recomendações, que enfatizaram a expansão da Estratégia Saúde da Família; a ampliação do acesso à APS; a formação de profissionais para atuação multidisciplinar na APS; a alocação de tecnologias para garantir resolutividade na APS; o aprimoramento da regulação/coordenação de serviços para fortalecer a APS como elemento estruturante do SUS; estrutura e financiamento; recursos humanos, provimento de profissionais, apoio e estímulo às equipes; produção e divulgação de conhecimento; transparência nas ações da APS; e o papel mediador da APS no sistema de saúde.

**Conclusões.:**

Os achados reforçam a ESF como melhor modelo para garantir uma APS forte no SUS, aliada a políticas que priorizem os atributos essenciais da APS, sobretudo pela inovação em tecnologias assistenciais, de gestão e de comunicação.

## Abstract (en)

**Objective.:**

To formulate strategic recommendations to strengthen primary health care (PHC) in Brazil’s Unified Health System (SUS) based on expert consultation.

**Method.:**

The present qualitative study, developed from March to August, 2018, included administration of an open questionnaire followed by consensus building among 20 participants representing the five Brazilian regions, selected using the criterion of recognized professional expertise in the field of PHC. Participants answered an online questionnaire created by the authors. The findings were systematized as recommendations, which were submitted for priority ranking by the expert group using a one-round Delphi technique. The final recommendations were discussed in a face-to-face workshop.

**Results.:**

Of 20 experts, 18 answered the open questionnaire, generating 84 themes for analysis, which were systematized into 44 proposals. Evaluation of these proposals resulted in 20 recommendations, emphasizing expansion of the Family Health Strategy (FHS); enhanced access to PHC; training of professionals for multidisciplinary work in the PHC setting; allocation of technologies to ensure PHC resolvability; improvement of regulation/coordination of services to strengthen a foundational role of PHC in the SUS; human resources, provision of professionals, and support/stimulus for teams; production and dissemination of knowledge; transparency in PHC initiatives; and mediating role of PHC in the healthcare system.

**Conclusions.:**

The findings support the FHS as the best model to ensure a strong PHC in the SUS, combined with policies that prioritize essential PHC attributes, especially through innovation in care, management, and communication technologies.

## RESUMEN

**Objetivo.:**

Formular recomendaciones estratégicas para fortalecer la atención primaria de salud (APS) en el Sistema Único de Salud (SUS) en Brasil a partir de una consulta a expertos.

**Método.:**

Este estudio cualitativo, realizado entre marzo y agosto del 2018, consistió en emplear un cuestionario abierto y lograr consenso entre 20 participantes representativos de las cinco macrorregiones brasileñas, seleccionados por su reconocida experiencia profesional en la APS. Los participantes respondieron 20 preguntas abiertas en un cuestionario en línea elaborado por los investigadores. Los hallazgos se sistematizaron en forma de recomendaciones, sometidas por el grupo de expertos a una evaluación de prioridades con la metodología Delfos en una sola ronda. Las recomendaciones finales se debatieron en un taller presencial.

**Resultados.:**

Dieciocho de los 20 especialistas respondieron al cuestionario abierto, que produjo 84 temas sistematizados en 44 propuestas. Después de la evaluación, se formularon 20 recomendaciones, en las cuales se acentuaron la expansión de la estrategia de salud de la familia; la ampliación del acceso a la APS; la formación de profesionales para el trabajo multidisciplinario en la APS; la asignación de tecnologías para garantizar la resolutividad en la APS; el perfeccionamiento de la regulación y la coordinación de servicios para fortalecer la APS como elemento estructurante del SUS; la estructura y el financiamiento; los recursos humanos, la dotación de profesionales, el apoyo y el estímulo a los equipos; la producción y divulgación del conocimiento; la transparencia en las actividades de APS; y la función mediadora de la APS en el sistema de atención de salud.

**Conclusiones.:**

Los hallazgos refuerzan la estrategia de salud de la familia como el mejor modelo para garantizar una APS fuerte en el SUS, vinculada a políticas que prioricen los atributos esenciales de la APS, sobre todo por medio de innovación en materia de tecnologías asistenciales, de gestión y de comunicación.

A atenção primária à saúde (APS) é considerada a principal e mais adequada forma de acesso das pessoas ao sistema de saúde, estando diretamente associada a uma distribuição mais equitativa da saúde entre populações (1). No Brasil, desde a implantação do Sistema Único de Saúde (SUS), que segue princípios de universalidade, integralidade e equidade, estabelecidos na Constituição Federal de 1988 (2), avanços consistentes foram feitos em direção à cobertura universal em saúde (3), especialmente após o estabelecimento da Estratégia Saúde da Família (ESF) como política nacional para implantação da APS (4). De 1998 a 2018, o número de equipes de ESF cresceu de 2 mil para 43 mil, passando a cobrir, potencialmente, cerca de 130 milhões de pessoas, o que corresponde a cerca de 62,5% da população brasileira (5). O aumento da cobertura de ESF está associado a melhorias no uso de serviços e nos resultados em saúde (6), com redução de internações por condições sensíveis à atenção primária (7) e de mortes por causas preveníveis (8). Além disso, possibilitou uma queda na mortalidade infantil em todas as regiões do país (9), o que beneficiou populações mais vulneráveis e reduziu iniquidades (10).

Entretanto, apesar dos resultados alcançados, estudos apontam que a expansão de cobertura de APS no Brasil enfrenta barreiras associadas a fatores como escassez de profissionais médicos, restrições orçamentárias, localizações remotas e maior cobertura populacional por planos privados de saúde (11, 12). Além disso, a heterogeneidade na qualidade da atenção à saúde prestada nos serviços de APS pelos municípios brasileiros (13, 14) persiste como um dos entraves para ampliar a capacidade de resposta a novos e antigos problemas de saúde que compõem o perfil sanitário brasileiro, caracterizado pela alta carga de doenças crônico-degenerativas, agravos de saúde mental, distúrbios alimentares, além de manutenção de doenças infecciosas e aumento na incidência de mortes violentas (15).

A busca por expandir a cobertura e ampliar a qualidade de serviços de APS no Brasil, seguindo os atributos essenciais definidos por Starfield (acesso de primeiro contato, longitudinalidade, integralidade e coordenação) (16), exige capacidade contínua de inovação na formulação e implantação de políticas, modelos e práticas em saúde no SUS. Essa necessidade foi reforçada diante do contexto de crise econômica e política que o país atravessa desde 2014, seguido pela introdução de políticas de austeridade de longo prazo em 2016 (17). Após um período de expansão no gasto em saúde no Brasil, ocorrido de 2000 a 2015 (de 7,0% para 9,1% do produto interno bruto), que permitiu promover aumentos progressivos do gasto público em saúde, houve, a partir de 2015, uma inversão de tendência, com queda no gasto governamental *per capita* em saúde (17).

Por outro lado, há necessidade de aumentar a eficiência do gasto em saúde, a fim de garantir que novos recursos signifiquem respostas efetivas às necessidades em saúde da população e não, apenas, mais gasto sem resultados concretos. Estudos do Banco Mundial apontaram oportunidades para aperfeiçoar a gestão do SUS e aumentar a eficiência no uso de recursos públicos. Entre as propostas, sugeriram-se a expansão da cobertura de APS e o fortalecimento de seu papel de porta de entrada do sistema de saúde; o desenvolvimento de redes integradas de atenção à saúde; e a implantação de novos arranjos de governança e gestão para aumentar a autonomia, a flexibilidade e a eficiência de provedores (18, 19). Além disso, verifica-se que a revolução em curso no campo de tecnologia de informação e comunicação (20, 21) poderá favorecer a ampliação em larga escala dos conceitos e princípios da APS, promovendo o acesso a serviços de qualidade e reduzindo custos operacionais desnecessários (22).

Reconhecendo que o Brasil se consolidou como um polo privilegiado para pesquisa em APS – dada a riqueza de experiências desenvolvidas no país nesse âmbito da atenção à saúde, assim como no ensino e pesquisa e gestão do sistema de saúde –, e levando em conta o cenário de avanços, desafios, ameaças e oportunidades, a representação da Organização Pan-Americana da Saúde (OPAS) no Brasil propôs o projeto “APS Forte”, integrante da Agenda OPAS “30 anos de SUS – que SUS para 2030?” (23). O projeto buscou elaborar recomendações, cientificamente embasadas e aplicáveis na prática, para fortalecer a APS no Brasil. No contexto desse projeto, o objetivo do presente artigo é apresentar o consenso de um grupo de especialistas quanto às ações necessárias para fortalecer a APS no âmbito do SUS.

## MATERIAIS E MÉTODOS

Com o objetivo de explorar a opinião coletiva de um grupo de especialistas brasileiros em APS a respeito dos desafios, das barreiras e das soluções para produzir uma “APS Forte” no Brasil, o presente estudo usou metodologia qualitativa, combinando a aplicação de questionário aberto e a busca de consenso entre os participantes por meio do método Delphi (24), em rodada única, finalizado por discussão presencial.

A pesquisa ocorreu de março a agosto de 2018 e foi aprovada pelo comitê de ética da OPAS. Todos os participantes assinaram termo de consentimento livre e esclarecido permitindo o uso das informações coletadas.

### Participantes

A seleção dos participantes da pesquisa foi definida pelos pesquisadores, buscando abranger pessoas das cinco macrorregiões do país, com conhecimento e experiência relevantes para responder às perguntas de interesse. Dessa maneira, foram considerados como critérios de *expertise*: a experiência anterior ou atual em cargos na administração do SUS (Ministério da Saúde, secretaria estadual ou municipal de saúde) e da saúde suplementar; e pelo menos 10 anos de atuação na assistência, docência e pesquisa em APS, com publicações relevantes na área.

Ao total, 20 pessoas foram convidadas para participar da pesquisa. O tamanho da amostra foi definido por conveniência. O pesquisador principal do estudo (RT) entrou em contato por via telefônica e, em seguida, enviou *e-mail* institucional da OPAS-Brasil para todos os participantes, a fim de formalizar o convite. Em ambas as oportunidades, foram informados os objetivos, os métodos e as informações gerais do estudo, incluindo o cronograma proposto para o desenvolvimento da pesquisa.

### Questionário aberto

Para a elaboração do questionário aberto, um dos pesquisadores (AM) realizou pesquisa na literatura. Entre os materiais identificados, selecionou-se o documento *Primary Care Evaluation Tool* (PCET), elaborado pelo escritório regional europeu da OMS (25), como referência para a preparação do questionário. Os itens foram sistematizados na forma de tópicos. Em seguida, outro pesquisador (EH) preparou as 20 questões que compuseram o questionário (tabela 1), abordando os seguintes temas: diretrizes organizacionais e modelo assistencial; rede de atenção à saúde; estrutura e recursos tecnológicos; recursos humanos; recursos financeiros; governança; participação social; e métodos de monitoramento e de avaliação de resultados.

**TABELA 1.** Questionário APS Forte: levantamento de estratégias, ações e inovações viáveis e cientificamente embasadas, Brasil, 2018

Tema	Questão
Diretrizes organizacionais e modelo assistencial	1. Você identifica estratégias, ações ou inovações que possam evidenciar e disseminar a importância dos atributos essenciais da APS (acesso de primeiro contato, longitudinalidade, integralidade e coordenação) para a concepção de políticas e para a organização assistencial? Descreva:
	2. Você identifica ações, programas ou processos de trabalho frequentes na APS brasileira que são obstáculos para o fortalecimento da mesma? Se sim, descreva essas práticas e aponte estratégias, ações ou inovações que possam estimular as equipes a abandonar essas práticas.
	3. Você identifica estratégias, ações ou inovações que poderiam aumentar o acesso de primeiro contato aos serviços de APS, especialmente a consultas médicas? Descreva:
	4. Você identifica estratégias, ações ou inovações que poderiam mudar a forma de adscrição da clientela na APS, dando maior relevância a necessidades, especificidades e poder de escolha da população para além do critério unicamente territorial? Descreva:
	5. Você identifica estratégias, ações ou inovações que poderiam aumentar a resolutividade clínica dos serviços de APS? Descreva:
	6. Você identifica estratégias, ações ou inovações que poderiam ampliar a carteira de serviços na APS? Descreva:
Rede de atenção à saúde	7. Você identifica estratégias, ações ou inovações que possam facilitar a comunicação e a integração entre os serviços de APS e os demais pontos da rede de atenção? Descreva:
	8. Você identifica estratégias, ações ou inovações que possam fazer da APS a ordenadora de fato da rede de atenção? Descreva:
Recursos humanos	9. Você identifica estratégias, ações ou inovações que possam tornar adequada a oferta de recursos humanos para o fortalecimento da APS? Descreva:
	10. Você identifica estratégias, ações ou inovações de educação permanente capazes de dar sustentação a uma APS Forte? Descreva:
	11. Você identifica novas funções para os profissionais de saúde ou inclusão de novas categorias profissionais que sejam essenciais para fortalecer a APS? Descreva:
	12. Você identifica estratégias, ações ou inovações que possam estimular a oferta de outras atividades assistenciais por parte das profissões não médicas, com ênfase em enfermeiros, técnicos de enfermagem, agentes comunitários e farmacêuticos? Descreva:
	13. Que medidas poderiam ser introduzidas para melhorar a gestão do trabalho dos profissionais da APS, e que incentivos poderiam ser empregados? Descreva:
Estrutura e recursos tecnológicos dos serviços de APS	14. Você identifica estratégias, ações ou inovações que possam qualificar a estrutura física das Unidades Básicas de Saúde? Descreva:
	15. Você identifica estratégias, ações ou inovações que possam qualificar equipamentos e insumos das Unidades Básicas de Saúde? Descreva:
	16. Você identifica tecnologias que deveriam ser incorporadas aos serviços de APS? Se sim, descreva essas tecnologias, assim como estratégias, ações ou inovações de efetividade comprovada que possam aumentar a incorporação tecnológica:
	17. Você identifica estratégias, ações ou inovações que qualificam o manejo das condições crônicas? Descreva:
Participação social	18. Você identifica estratégias, ações ou inovações que democratizem, ampliem e tornem mais efetivos os dispositivos de participação social? Descreva:
Monitoramento e avaliação de resultados	19. Você identifica estratégias, ações ou inovações relacionadas ao monitoramento e avaliação de processos e de resultados na APS? Descreva:
Outras fragilidades e barreiras	20. Agora que você já fez sugestões de estratégias, ações e inovações para o fortalecimento da APS no SUS, cite: a) outros desafios e fragilidades que não foram explorados no item de Contextualização deste documento; b) os principais fatores que podem impedir a concretização das ações e inovações propostas para o fortalecimento da APS no SUS.

Antes de ser submetido aos participantes, o questionário foi testado para identificar ambiguidades, pontos obscuros e, caso necessário, ajustar as perguntas. Em seguida, o instrumento foi disponibilizado *on-line* através da ferramenta SurveyMonkey. Na página inicial do questionário constavam esclarecimentos sobre o projeto e seus objetivos e um termo de consentimento informado. O acesso às perguntas só era possível caso houvesse concordância com a participação. As respostas eram anônimas, porém solicitou-se identificação do segmento de atuação do participante. Exceto pelo consentimento informado, a resposta às perguntas não era obrigatória. Não foi estabelecido limite de palavras para as respostas. O questionário permaneceu disponível aos participantes de 20 a 30 de abril de 2018.

### Análise das respostas

O conjunto de respostas individuais a cada pergunta do questionário aberto foi exportado em PDF e em Excel. Todos os pesquisadores participaram da análise das respostas individuais.

Incialmente, dois pesquisadores (WMC e CB) analisaram as respostas individuais, de forma independente, utilizando técnica de análise de conteúdo para categorizar unidades de texto para agrupamento por similaridade em grandes categorias (diretrizes organizacionais, rede de atenção à saúde, recursos humanos, estrutura e recursos tecnológicos dos serviços de APS, participação social, monitoramento e avaliação de resultados e outras fragilidades e barreiras). Em seguida, as propostas consolidadas foram contrastadas e compiladas em uma lista, sendo consultados os outros três pesquisadores caso não houvesse consenso quanto a alguma proposta.

Ao final desse processo, foram geradas 44 propostas de recomendação para fortalecimento da APS no Brasil, que foram submetidas para avaliação dos participantes em formato Delphi.

### Técnica Delphi e reunião de consenso

As propostas consolidadas a partir da análise das respostas do questionário aberto foram disponibilizadas para validação e definição de prioridades pelo grupo de participantes. Para isso, um novo questionário estruturado foi disponibilizado na ferramenta SurveyMonkey em maio de 2018. Nessa etapa, os participantes precisavam obrigatoriamente responder todas as perguntas. Para cada proposta, apresentavam-se as seguintes alternativas: não incluir; incluir com prioridade alta; incluir com prioridade média; ou incluir com prioridade baixa.

Finalmente, apresentaram-se as propostas ranqueadas de acordo com seu grau de prioridade em reunião presencial com participantes e pesquisadores, para consenso quanto à lista definitiva de propostas e a redação final. A reunião ocorreu em uma oficina de trabalho que durou 8 horas, realizada no pré-Congresso Brasileiro de Saúde Coletiva (ABRASCO) em 25 julho de 2018.

As propostas foram apresentadas da seguinte forma, conforme material disponível *on-line* (26):

- atributos da APS com prioridade alta em ≥70% das respostas do Delphi;
- atributos da APS com prioridade alta em <70% das respostas do Delphi;
- macrocondições sistêmicas com prioridade alta ≥70% das respostas do Delphi;
- macrocondições sistêmicas com prioridade alta <70% das respostas do Delphi;
- itens com exclusão sugerida por >15% dos participantes.

As recomendações aprovadas em consenso na reunião presencial foram, finalmente, enviadas por e-mail aos participantes e validadas em seu formato final.

## RESULTADOS

Dos 20 convidados, 18 (90%) responderam o questionário individual, 16 (80%) participaram do Delphi e 14 (70%) participaram da reunião de consenso. Dos participantes, 56% eram gestores ou ex-gestores em saúde, e 50% eram professores e/ou pesquisadores de universidade. Também houve representação, em menor grau (11,1%), de profissionais vinculados ao setor privado de saúde (tabela 2).

**TABELA 2.** Setor de atuação dos respondentes do questionário aberto para identificar estratégias de fortalecimento da APS no SUS, 2018

Setor	No.	%
Gestor ou ex-gestor em saúde	10	55,6
Federal (Ministério da Saúde)	6	
Estadual (Distrito Federal e Amazonas)	2	
Municipal (Rio de Janeiro e Curitiba)	2	
Setor privado	2	11,1
UNIMED	1	
AMIL	1	
Professor/pesquisador de universidade	9	50,0
Grupo Hospitalar Conceição	1	
Fiocruz – Fundação Oswaldo Cruz	1	
UFPB – Universidade Federal da Paraíba	1	
UFPEL – Universidade Federal de Pelotas	1	
UFRGS – Universidade Federal do Rio Grande do Sul	1	
UNB – Universidade Nacional de Brasília	1	
UNICAMP – Universidade Estadual de Campinas	2	
USP – Universidade de São Paulo	1	
Outros	4	22,2
COFEN - Conselho Federal de Enfermagem	1	
OPAS – Organização Pan-Americana da Saúde	1	
SBMFC – Sociedade Brasileira de Medicina de Família e Comunidade	2	

a Até 2 respostas por participante.

O total de respostas aos questionários individuais, após agrupamento por afinidade, gerou 86 itens temáticos. Esse material, após sistematização, resultou em 44 propostas, que foram discutidas pelos participantes para análise de prioridades na rodada Delphi. Esse produto (26) foi apresentado na reunião de consenso. Ao final da reunião de consenso, foram aprovadas 20 recomendações para uma APS forte no SUS no Brasil (tabela 3).

**TABELA 3.** Recomendações para fortalecer a atenção primária à saúde no Brasil

Tema	Recomendação
Expansão/consolidação da ESF	1. Ampliar e consolidar a ESF com ênfase nos atributos essenciais da APS.
Ampliação do acesso à APS	2. Ampliar formas de acesso à APS, como acesso avançado, acesso não presencial e horário estendido, além de incorporar ferramentas digitais para comunicação não presencial entre equipe e pessoas (por exemplo: marcação não presencial de consultas, teleconsulta, e-mail, aplicativos).
	3. Qualificar a adscrição de pessoas às equipes de APS, utilizando quantitativo populacional e critérios de adscrição complementares aos critérios territoriais, epidemiológicos e de vulnerabilidade social, como o uso de lista de pacientes.
	4. Ofertar ações e serviços de saúde de acordo com as necessidades da população, formulando uma carteira de serviços com garantia dos recursos – insumos, equipamentos, entre outros – e das competências profissionais que garantam a plena execução da carteira.
Formação de profissionais para atuação multidisciplinar na APS	5. Ampliar a atuação clínico-assistencial de todas as categorias profissionais das equipes de APS, com a utilização de protocolos multiprofissionais baseados na melhor evidência cientifica disponível.
	6. Qualificar habilidades dos profissionais de APS em relação a comunicação e tecnologia do cuidado (por exemplo, entrevista motivacional, plano de cuidados e autocuidados).
Alocação de tecnologias para garantir resolutividade da APS	7. Promover adensamento tecnológico orientado pela prevenção quaternária na APS, utilizando tecnologias de informação e equipamentos diagnósticos e terapêuticos (por exemplo: ultrassonografia, eletrocardiograma) de forma presencial ou a distância.
	8. Informatizar as unidades básicas de saúde, a rede assistencial e os complexos reguladores; disponibilizar registro eletrônico em saúde com informações tanto do sistema público como privado, de forma unívoca, permitindo às pessoas o deslocamento físico entre os pontos assistenciais, sem barreiras informacionais.
Aprimoramento da regulação/coordenação de serviços para fortalecer a APS como elemento estruturante do SUS	9. Desenvolver sistema de regulação centrado na APS, com ênfase em tecnologias da informação e comunicação e protocolos clínicos de regulação, com qualificação do processo de referência e contrarreferência.
Estrutura e financiamento	10. Aumentar o financiamento da APS até atingir níveis adequados e suficientes.
	11. Garantir estrutura física e tecnológica adequadas, com ambiência, conforto e fornecimento adequado de insumos para o funcionamento das unidades básicas de saúde.
Recursos humanos, provimento de profissionais, apoio e estímulo às equipes	12. Planejar a oferta de recursos humanos para a APS e elaborar plano de formação profissional com ênfase nas especificidades da atenção primária (por exemplo, médico de família e comunidade, enfermeiro de família e comunidade).
	13. Ter estratégia permanente e sustentável de provimento de médicos para APS em áreas com alta taxa de rotatividade profissional ou dificuldade de alocação de médicos.
	14. Promover apoio assistencial às equipes de APS (por exemplo: cuidado compartilhado, interconsultas, telessaúde NASF, matriciamento), de forma presencial ou a distância.
	15. Promover, monitorar e avaliar a qualidade da atuação das equipes de APS, quanto a princípios, atributos, diretrizes, objetivos, metas e resultados, com estabelecimento de mecanismos de remuneração e incentivos por desempenho.
	16. Estimular e formar lideranças em APS no âmbito da gestão.
Produção e divulgação de conhecimento	17. Promover estratégias de defesa e fortalecimento da APS, incluindo produção de conhecimento científico e divulgação de experiências inovadoras e exitosas.
Conferir transparência às ações da APS	18. Reforçar a transparência das informações sobre saúde, facilitando o acesso da população a informações sobre as ações e os serviços de saúde (por exemplo: listas de espera, horários, serviços ofertados), com uso de tecnologia da informação e outros dispositivos de divulgação.
	19. Favorecer a participação das pessoas e a avaliação dos serviços pela incorporação de novos canais de escuta por meio de tecnologias de comunicação não presenciais, ouvidoria, entre outros.
Papel mediador da APS	20. Incentivar o papel mediador da APS frente a ações intersetoriais e à participação das pessoas para incidir na determinação social, promover a saúde e reduzir as desigualdades.

## DISCUSSÃO

Os 40 anos de Alma-Ata e os 30 anos do SUS serviram como oportunidade para analisar avanços, desafios, ameaças e oportunidades referentes aos sistemas de saúde no Brasil e no mundo. Experiências globais, com destaque para os 70 anos do Sistema Nacional de Saúde inglês (27), consolidaram um consenso de que a APS é o mais apropriado, seguro, custo-efetivo e sustentável modo de organizar sistemas de saúde, como também é o meio mais adequado para os países atingirem a cobertura universal em saúde (28). Entretanto, mesmo países com sistemas de saúde bem estabelecidos enfrentam desafios quanto ao modo de funcionamento da APS no século XXI (29-34).

Os achados no presente estudo corroboram o que diz a literatura internacional. Segundo os especialistas consultados, a ESF é o modelo mais adequado para o funcionamento da APS no país, devendo, portanto, ser expandido e consolidado (Recomendação 1). Porém, para que a ESF tenha capacidade de inovar, aprimorando a capacidade de resposta aos problemas de saúde contemporâneos, é preciso investimento na formação profissional, na incorporação racional de tecnologias, incluindo tecnologias de informação e comunicação, e na criação de adequadas condições de trabalho para as equipes multiprofissionais.

Dessa forma, um dos pontos principais das recomendações está no reconhecimento de que a produção de saúde se faz entre pessoas e de que é preciso aprimorar o modo de relação estabelecido entre os serviços de APS com os seus usuários. É essencial que os serviços sejam facilmente acessíveis à população, que o usuário esteja no centro da atenção e que a orientação às necessidades em saúde das comunidades seja a base da organização dos serviços.

Para tanto, recomenda-se estabelecer mecanismos de acesso avançado (agendar consultas no mesmo dia ou em até 48 horas após o contato do usuário com o serviço de saúde), acesso não presencial e ampliação do horário de funcionamento das unidades de saúde, além da criação de meios de comunicação não presencial entre equipe e pessoas (*on-line*, e-mail e telefone) (Recomendação 2); adscrição complementar aos critérios territoriais, epidemiológicos e de vulnerabilidade social, como o uso de lista de pacientes (Recomendação 3); e modelagem de carteiras de serviços que contemplem o conjunto de necessidades de saúde apresentadas pela população (Recomendação 4).

A ampliação do acesso a serviços de APS, por outro lado, deve ser combinada ao aprimoramento da performance clínica das equipes de saúde para se tornar mais resolutiva. Nesse aspecto, as recomendações apontam para a necessidade de fortalecer competências clínico-assistenciais de todas as categorias profissionais que atuam na APS, enfatizando o uso de protocolos multiprofissionais baseados na melhor evidência científica disponível (Recomendação 5); o desenvolvimento de habilidades de comunicação e tecnologia do cuidado (Recomendação 6), combinando a incorporação de tecnologias de informação e equipamentos diagnósticos e terapêuticos (Recomendação 7); e a garantia de condições de trabalho adequadas, com estrutura física e fornecimento de insumos (Recomendação 11).

Outro aspecto destacado diz respeito à criação de estratégias para incentivar o trabalho colaborativo na atenção primária. É necessário integrar a APS com os demais níveis do sistema de saúde em redes assistenciais inseridas em regiões de saúde. Para tanto, o estabelecimento de complexos reguladores e a informatização das unidades de saúde, com disponibilização de informações clínicas integradas, dotada de informações tanto do sistema público como privado, são cruciais para permitir o deslocamento físico entre os pontos assistenciais, sem barreiras informacionais (Recomendação 8).

A regulação, portanto, deve estar centrada na APS, utilizando protocolos clínicos que permitam a qualificação do processo de referência e contrarreferência (Recomendação 9). O estabelecimento de suporte técnico especializado às equipes de APS, também denominado de apoio matricial, de forma presencial ou a distância (Recomendação 14), favorece a integração da APS com o sistema de saúde por dinamizar a comunicação entre equipes, contribuindo para aumento da capacidade resolutiva na APS.

A disponibilidade de equipes profissionais com adequada formação é uma das condições para a APS cumprir seu objetivo no sistema de saúde. Dessa maneira, as recomendações também apontam para a necessidade de planejar a oferta de recursos humanos para a APS, elaborando plano de formação profissional com ênfase nas especificidades desse campo de atuação (Recomendação 11), bem como dispor de estratégia permanente e sustentável de provimento de médicos para APS em áreas com dificuldade de alocação desse profissional (Recomendação 12).

Vale reforçar que as recomendações evidenciam a necessidade de financiamento em níveis adequados e sustentáveis (Recomendação 10). Embora muitos recursos possam ser otimizados com uma gestão eficiente, apropriada e com combate ao desperdício, é preciso reconhecer a possibilidade de crescimento contínuo da demanda por fatores epidemiológicos, como o envelhecimento populacional e a introdução de novas tecnologias e conhecimento, e, inclusive, pelo necessário aumento do acesso a parcelas ainda excluídas da população.

Por outro lado, o financiamento da APS deve estar articulado ao monitoramento e à avaliação da qualidade da atuação das equipes de APS quanto a princípios, atributos, diretrizes, objetivos, metas e resultados, com estabelecimento de mecanismos de remuneração e incentivos por desempenho (Recomendação 15).

Os especialistas identificam, ainda, a necessidade de estratégias de *advocacy* para a defesa e o fortalecimento da APS perante a sociedade e a comunidade científica. Para isso, recomendam o estímulo à formação de lideranças em APS (Recomendação 16) e à produção de conhecimento científico, juntamente com a divulgação de experiências inovadoras e exitosas (Recomendação 17).

Recomenda-se, também, o incentivo à divulgação de informações sobre APS, visando a dar transparência e facilitar o conhecimento da população sobre serviços oferecidos, como, por exemplo: listas de espera, horários e serviços ofertados (Recomendação 18), bem como resultados obtidos a partir das ações de saúde. Por isso, recomenda-se favorecer a participação das pessoas e a avaliação dos serviços pela incorporação de novos canais de escuta por meio de tecnologias de comunicação não-presenciais, ouvidoria, entre outros (Recomendação 19). Por fim, diante do reconhecimento da complexidade dos problemas de saúde contemporâneos, recomenda-se incentivar o papel mediador da APS frente a ações intersetoriais e à participação das pessoas para incidir na determinação social, promover a saúde e reduzir as iniquidades (Recomendação 20).

O presente estudo apresenta algumas limitações. Primeiro, apesar do esforço de tentar contemplar a realidade regional, a amostra de pesquisa não foi suficiente para retratar a diversidade de experiências do país, tampouco a representação dos diversos profissionais que atuam no âmbito da APS. Em segundo lugar, um maior número de participantes e de rodadas Delphi poderia enriquecer ainda mais o material.

Por outro lado, um dos pontos fortes do estudo foi reunir, de maneira inédita, um grupo altamente qualificado de especialistas brasileiros em APS, de diferentes espectros políticos, com distintas visões e variadas experiências, em um ambiente social altamente polarizado vivido no Brasil e no mundo. O conhecimento coletivo produzido nesse encontro permitiu elaborar recomendações cientificamente embasadas e aplicáveis na prática, que podem contribuir para o fortalecimento da APS.

Essas recomendações ganham ainda mais importância diante de um contexto político e econômico complexo, em que medidas de austeridade fiscal ameaçam o ideal da universalidade, que guiou, em grande parte, as políticas nacionais de saúde no Brasil até o presente momento.

## CONCLUSÃO

As recomendações apresentadas neste artigo, produzidas a partir do conhecimento coletivo de um grupo de especialistas brasileiros, podem ser de grande valia para gestores do SUS e para o desenho de políticas públicas para APS. Elas mostram um caminho claro e objetivo voltado para fortalecer a ESF e a APS como componente organizador do SUS. As recomendações também podem ser úteis para os países latino-americanos que desejem implementar políticas voltadas para o avanço do acesso e cobertura universal em saúde.

## Contribuição dos autores.

RT, EH, AM idealizaram o estudo. RT, AM, WMC, CB, EH elaboraram os questionários, revisaram e compilaram os dados, participaram da reunião presencial de consenso e redigiram e revisaram o artigo. Todos os autores aprovaram a versão final.

## Agradecimentos.

Os autores agradecem a Lisiane Hauser pelo apoio na elaboração dos questionários on-line. Agradecem ainda a André Luís Bonifácio de Carvalho, Carmen Lavras, Cesar Monte Serrat Titton, Charles Tocantins, Claunara Schilling Mendonça, Daniel Knupp Augusto, Daniel Ricardo Soranz Pinto, Eugênio Vilaça Mendes, Gilmara Lucia dos Santos, Gonzalo Vecina Neto, Gustavo Diniz Ferreira Gusso, Heider Aurelio Pinto, Heloiza Machado de Souza, Humberto Lucena Pereira da Fonseca, Ligia Giovanella, Luis Fernando Rolim Sampaio, Luiz Augusto Facchini, Nulvio Lermen Junior, Rubens Bedrikow, Silvia Maristela Pasa Takeda e Thiago Gomes da Trindade pelas contribuições essenciais para a construção destas 20 Recomendações.

## Financiamento.

O projeto teve apoio financeiro da Organização Pan-Americana da Saúde (OPAS).

## Declaração.

As opiniões expressas no manuscrito são de responsabilidade exclusiva dos autores e não refletem necessariamente a opinião ou política da RPSP/PAJPH ou da Organização Pan-Americana da Saúde (OPAS).
